# The Paramount Importance of a Thorough Dental Education, and the Necessity of Union among the Profession to Support the Existing Institutions for Its Dispensation

**Published:** 1844-03

**Authors:** James D. McCabe


### ARTICLE V.

*The Paramount Importance of a thorough Dental Education, and the necessity of union among the profession to support the existing Institutions for its Dispensation.* By James D. McCabe, D. D. S.

The present is emphatically a day of reform and improvement; *progress* is written upon every page of the world's present history, as it is turned to view by the hand of time. The arts and sciences are developing their vast resources, and approximating the goal of perfection—every department of life breathed upon by the pervading spirit, unites in the race after excellence. Old systems are being exploded, or else so strangely changed by the new life infused into them, as to be scarcely recognized in the new forms with which they appear. The age of iron is fast passing away, and the true golden age of the world begins to dawn around.

He, who like the Ephesian sleepers, has for the last quarter of a century indulged in a temporary oblivion, awakening now, would scarcely distinguish the theatre of his being, so much have the aspect of affairs been changed. Poetry, painting and music, have assumed their divinest forms. The exact sciences are brought to a perfection unknown before; literature rolls out its perennial streams, in fertilizing rills; the mechanic arts are rapidly abridging the consumption of labor, and united with science in the construction of the steam engine, has transformed the ocean into a highway, and made neighbors of the most distant nations. On no science has the hand of progress more forcibly and distinctly left its impress, than on medicine and its kindred branches. Its pulses now beat with vigorous health, and its healing miracles are all abroad over the face of the wide earth.

Its infinitely diversified fields of investigation and experiment, have been explored by the most profound, and boldest minds, that ever moved along the path of scientific discovery, and as it now exists in the sunlight of French genius, softened and refined by the profound judgment of the English, and inventive spirit of the American schools, it is confessedly the most perfectly cultivated science known to the world. It is true it has its *quacks* and its *empirics*, its Homœopathy, and its Thomsonianism, but these excrescences which have grown out upon its healthy and vigorous trunk, only show the strength of the system which has driven them to the surface.

In dentistry, that department or branch of the art of healing, which has for the circle of its manipulations and labors, the treatment of diseases of the teeth and mouth, the hand of improvement and progress is equally apparent; and we have only to contrast its present resources and condition with what they were a few years back, to set this fact most lucidly before our minds in all its interesting aspects.

The metaphysician, has declared that the splendid figure of *Venus de Medici*, with its perfect symmetry and beautiful expression, existed in the unshapen marble of the quarry, ere the chisel of the statuary had removed the obstructions that concealed it, or developed its beauties. Thus with dentistry, its perfect laws—its exact and definite proportions—its powers and its capabilities, were all there, but it has required the hand of a *Pygmalion* to remove the rubbish, and display its latent excellence; it stands before us now in the dignity of its high claims, arrayed in the habiliments of science, a potent agent in the removal of many of the ills to which flesh is heir.

The night of its darkness and disgrace, we trust, has passed, and the day now beaming around us, lighted with unnumbered facilities for its cultivation, as a distinct science, will, we doubt not, unite its disciples throughout the length and breadth of the land, in vigorous efforts to develope its capabilities for usefulness.

The obligation resting upon each member of the profession to contribute his individual aid, and influence to the promotion of "a consummation *so* devoutly to be wished" as that stated above, is not, we humbly conceive, sufficiently considered by our professional brethren, and it has been one principal object in the hasty composition of this essay, to insist upon *the paramount importance of a thorough dental education, and the necessity of union among the profession to support the existing institutions for its dispensation.*

The reasonableness of the first branch of this proposition will be apparent to every considerate mind. A strict sense of moral justice, recognized before written codes were known, has always demanded proper preparation for any and every pursuit, and the more extensive the responsibility assumed in the pursuit, the more thorough the preparation that is demanded. The merest mechanic does not set up for himself until he has at least obtained a partial insight into the principles of his art; if he does, he becomes the butt of the craft with whom he seeks to associate.

What would be thought of the blacksmith and his capabilities, who, without further preparation, should abandon his *anvil*, and undertake to repair the delicate machinery of a watch. And yet how many hundreds rush into the practice of dentistry, as perfectly inadequate to its responsible duties, as would be the ignorant blacksmith to the repairs and accurate regulation of the chronometer. This rashness too is enhanced by the superior responsibilities of the vocation they assume.

The various organs of the human system are so mysteriously connected by a chain of sympathies and dependencies, that

> "Whatever link you strike,
> Tenth or ten-thousandth breaks the chain alike."

The teeth are of the highest importance to health, and constitute a link, that broken or touched too roughly, vibrates through the entire chain, and may lay the foundation of deep and incurable malady, in some distant organ. The preparation, therefore, for the treatment of their diseases, must be laid in an acquaintance with the laws of the system, of which they are a part. Anatomical, physiological, and sound pathological information is indispensible, and it is not the custom of the truly scientific mind to inquire how slight an acquaintance with these subjects *will do*, it will press on to the highest point of perfection, and never rest satisfied until the loftiest summit of excellence is attained.

The various systems and tissues, are more or less affected by every species of dental disease, from the accretions of salivary calculus, to the thrilling, racking, torturing throbs of acute odontalgia, and this too from the period when the teeth are first developed in their deciduous forms, until like the stunted oaks of the sand beach, they appear worn by attrition, few and far between, in the gums of old age and decrepitude. There are also various affections of the gums and membranes of the mouth, some local, and others the result of constitutional diathesis, which scientific skill can alone successfully treat, and in the management of which is required all the lights of sound judgment and surgical experience. These, and a thousand considerations that might be presented, urge the importance of sound and thorough dental education, for if its capacity to bless and improve mankind, is to be fully tested, it must be done by qualifying its practitioners to assume its responsibilities, and apply its acknowledged rules of practice, extended as they inevitably will be, by sound judgment and patient induction.

These obligations acknowledged, prove the importance of the second branch of the proposition, viz. *The necessity of union among the profession, to support the existing institutions for its dispensation.*

That there are some members of the profession who are in their feelings and conduct, hostile to these institutions, is a painful fact. With less qualifications for practice, than hundreds of their brethren, they have by dint of impudence, or a smuggling process, secured an M. D. to their names, and even while they are earning their bread by the manipulations of our art, are influenced by a sort of ignorant pride, to refuse to hold professional intercourse with any dentist who has not an M. D. to *his* name; and they seek to destroy the labor of the truly meritorious men of the profession, by speaking lightly of the agencies they have established for education, or else damning them with faint praise. I have been amused at some anecdotes I have heard of some of these *learned* gentlemen, and especially one, who lays claim to great consideration as a dentist, because *he attended in* Paris *the lectures of Valpace, on Midwifery.* There are others too, who from indolence, are enemies to the efforts for the diffusion of dental knowledge. These institutions are therefore dependent upon the support of the *intelligent* members of the profession, those who feel an *honest* pride in professional excellence.

The American Society of Dental Surgeons, and the Virginia Society, (and others which may be formed in the several states,) present a means to unite the profession, to bring them together, and inspire a proper zeal in the elevation of their art.

The Journal, as the record of the transactions of all, should have a place in the library of every practitioner, and by the simple operation of these facilities, a large share of what is required, will be accomplished, but the crowning act, will devolve upon "The Baltimore College of Dental Surgeons." This last institution, presents itself with strong claims upon the profession, as *the only dental college* in the world!

It can, and will be liberally supported if the members of the dental profession will generally agree to abandon a course of action—once proper and right in consequence of the condition of the profession, now no longer so—I allude to the practice of keeping students in the office twelve or eighteen months, and then sending them forth to practice, with only *their* certificate of competency. Let them send the young men to the college, and impress upon their minds, that the diploma of an honest and impartial faculty, or board of examiners, is the best *prima facia* evidence of skill and worthiness to practice; not that the parchment *per se*, is a qualification, but that it is evidence of respect for his profession on his part, and that he has made himself familiar with those great principles of science, which rightly practised, will ensure professional distinction.

It is true, there may be instances in which merit may not have the means to obtain these advantages, and must content itself with the office. Whenever this is the case, there are the examining boards of the societies, authorised to grant certificates of competency, and having obtained these, the students should seek by honest and patient industry, the necessary means to enable him to reap the advantages of at least one college course. The determination should be to sustain the college at all hazards, even if the societies are compelled to aid by an appropriation of their funds.

I have witnessed the salutary influence of societies, in bringing those into union who would otherwise, have remained at a perpetual distance, but never more clearly than in the institution of the American and Virginia Societies of Dentistry. The operation of the latter, has particularly fallen under my notice; from comparative strangers, the members have been united as brothers, improper rivalries have given place to noble emulations, a free and full interchange of professional courtesies are had, and a generous spirit exists to advance each other's interests, and build up each other's reputation. If this were all that is to be gained, we should be amply rewarded for all the toil and labor we are required to expend.

From the hurried statements before us, we conclude it to be the duty of each member of the profession, to cast his influence into the scale of effort, and with generous ambition, contest the palm of excellence with those who have elevated American dentistry in professional knowledge, and artistical skill, high above the efforts of transatlantic professors. The pride of England and of France, must bow to the American galaxy, composed of such men as Hayden, Harris, Parmly, Brown, and others, who have toiled up the steep of professional eminence, and have kindled their fires upon its summit as beacons to the world.

